# A Comparison of Drug Transport in Pulmonary Absorption Models: Isolated Perfused rat Lungs, Respiratory Epithelial Cell Lines and Primary Cell Culture

**DOI:** 10.1007/s11095-017-2251-y

**Published:** 2017-09-18

**Authors:** Cynthia Bosquillon, Michaela Madlova, Nilesh Patel, Nicola Clear, Ben Forbes

**Affiliations:** 1 School of Pharmacy, University of Nottingham, Boots Science Building, University Park, Nottingham, NG7 2RD UK; 2 King′s College London, Pharmaceutical Science Division, Franklin-Wilkins Building, 150 Stamford Street, London, SE1 9NH UK; 3 Faculty of Pharmacy, Charles University in Prague, Hradec Kralove, Czech Republic; 4 School of Pharmacy, University of Reading, Whiteknights, Reading, RG6 6AP UK; 5 Pfizer R&D, Sandwich, Kent CT13 9NJ UK

**Keywords:** 16HBE14o-, biopharmaceutics, calu-3, inhalation, isolated perfused lungs (IPL), NHBE, permeability, pulmonary

## Abstract

**Purpose:**

To evaluate the ability of human airway epithelial cell layers and a simple rat isolated perfused lung (IPL) model to predict pulmonary drug absorption in rats *in vivo*.

**Method:**

The permeability of seven compounds selected to possess a range of lipophilicity was measured in two airway cell lines (Calu-3 and 16HBE14o-), in normal human bronchial epithelial (NHBE) cells and using a simple isolated perfused lungs (IPL) technique. Data from the cell layers and *ex vivo* lungs were compared to published absorption rates from rat lungs measured *in vivo*.

**Results:**

A strong relationship was observed between the logarithm of the *in vivo* absorption half-life and the absorption half-life in the IPL (*r* = 0.97; excluding formoterol). Good log-linear relationships were also found between the apparent first-order absorption rate *in vivo* and cell layer permeability with correlation coefficients of 0.92, 0.93, 0.91 in Calu-3, 16HBE14o- and NHBE cells, respectively.

**Conclusion:**

The simple IPL technique provided a good prediction of drug absorption from the lungs, making it a useful method for empirical screening of drug absorption in the lungs. Permeability measurements were similar in all the respiratory epithelial cell models evaluated, with Calu-3 having the advantage for routine permeability screening purposes of being readily availability, robust and easy to culture.

## Introduction

The rate and extent of absorption of inhaled drugs are determined by the relative rates of the different clearance mechanisms that operate in the lungs [1–3]. Clearance by absorptive transfer from the lung lumen is predominately controlled by the epithelial permeability of free (unbound) drug. Although *in vitro* epithelial cell culture [4] and *ex vivo* lung methods [5] are available to screen the permeability of drug candidates for development as orally inhaled products, there is no standard experimental method for measuring drug permeability or predicting lung absorption [2]. As drug permeability in the lungs has been proposed recently to be a key factor in a biopharmaceutical classification system being developed for inhaled compounds (iBCS; [6]), the validation of screening techniques for predicting absorptive clearance from the lungs is of high importance.

The use of human epithelial cell lines as models for drug transport in the lungs is limited to airway cell lines because established and newer alveolar epithelial cell lines, (A549 [7] and TTI [8] cell lines, respectively) have proved unsuitable as models for screening drug permeability as they do not form cell layers with barrier properties representative of the lung epithelium [4,7,8]. The potential for the human airway epithelial cell lines Calu-3 and 16HBE14o- to be cultured as drug absorption models was recognized in the late 1990’s [9,10], and they have become the pre-eminent human respiratory epithelial cell lines for measuring drug permeability. Methods have been optimized for culturing 16HBE14o- cells [11,12] and Calu-3 cells [13–15] such that they exhibit epithelial barrier-like properties, and the permeability of a wide variety of compounds has been measured in these cell layers in different laboratories [4]. Furthermore, the drug permeability in Calu-3 [16] and 16HBE14o- cells [17] has been correlated with absorption from the lungs *in vivo* and *ex vivo*, respectively. Although these models have been evaluated individually, to date the permeability of solutes in the two cell lines has not been compared directly or matched to permeability in primary normal human bronchial epithelial cells.

Isolated perfused rat lungs (IPL) provide an *ex vivo* intact organ model with many applications for evaluating pulmonary biopharmaceutics [5], including estimation of drug absorption. The ability of an IPL model to predict drug absorption from the lungs has been reported by Tronde and co-workers [18,19]. However, most IPL methods use bespoke apparatus to preserve and monitor the mechanical functioning of the lungs *ex vivo*, e.g. negative pressure ventilation, monitoring of perfusion pressure and airway compliance together with custom spray or aerosol delivery systems to distribute drugs as evenly and as deeply as possible throughout the lung. This complexity has been a barrier to widespread adoption of the IPL as a biopharmaceutical screening technique [5]. We investigated whether drug permeability in the lungs can be measured using a much simpler IPL in which drug solution is instilled into statically inflated lungs from which drug transfer into vascular perfusate is measured.

This study was designed to provide a systematic comparison of solute permeability in different human airway epithelial cell culture systems and a simple rat IPL, avoiding inter-laboratory variation. Model compounds were selected carefully to possess a range of log P and reported pulmonary absorption data in rats; these included two drugs licensed as inhaled medicines (Table I). Novel features of the study included, (i) measuring solute permeability in 16HBE14o- and Calu-3 models compared to NHBE cells to evaluate whether the measurements in the cell line monocultures were comparable to permeability in the more physiologically-based primary cell layers which feature both ciliated and goblet cells, (ii) measuring the absorptive transport of the same drugs in a deliberately simple IPL system to investigate whether this technically less demanding model could determine drug permeability similarly to more complex perfused rat lungs [19] and the cell models. Finally, the relationship between the experimental data and reported absorption of the same compounds from rat lungs *in vivo* was evaluated.

## Materials and Methods

### Chemicals and Reagents

Test compounds; [^3^H]-formoterol, [^3^H]-terbutaline, [^3^H]-metoprolol were purchased from Vitrax (Placentia, USA), [^3^H]-propranolol from Amersham (Amersham, UK), [^3^H]-imipramine from Perkin-Elmer (Bucks, UK) and [^14^C]-dextran 10 K from Sigma-Aldrich (Poole, UK). Paracellular markers; [^3^H]-mannitol and [^14^C]-mannitol were obtained from Sigma-Aldrich and Amersham (Amersham, UK), respectively. Ready Protein^+®^ scintillation cocktail was purchased from Beckman Coulter (High Wycombe, UK). Cell culture supports were obtained from Corning Costar (Corning, UK). All cell culture reagents and all other chemicals were obtained from Sigma-Aldrich (Poole, UK).

### Simple Isolated Perfused rat Lung Method

Eight-week old male Wistar rats were obtained from Harlan UK Ltd. (Oxon, Oxfordshire). They were fed with a SDS RM1(E) maintenance diet (Special Diets Services Ltd., Essex). They were maintained at 20–21°C and 45–60% humidity with a 12 h light/dark cycle. All procedures performed on these animals were in accordance with regulations and established guidelines and were reviewed and approved by an Institutional Animal Care and Use Committee or through an ethical review process.

Rats were sacrificed with a lethal injection of pentobarbital (130 mg/kg body weight). As soon as they were unconscious, rats were secured in a supine position on a board inclined at approximately 45°. A midline incision was made from the neck to the abdomen using a scalpel blade and the rat was exsanguinated by severing the main abdominal vessels. The trachea was exposed and carefully pierced through one wall with a 21 G needle. A 3 cm long cannula made of a polyethylene tubing (PolyE 240, Harvard Apparatus Ltd., Edenbridge, UK) mounted on a blunt 21 G needle was introduced into the trachea. This was securely tied with two suture threads (Silk black braid USP size 4.0, Harvard apparatus Ltd) and a 25 mm Dieffenbach’s bulldog artery clip (Scientific Laboratory Supplies Ltd., Nottingham, UK). The diaphragm was cut open, 0.5 mL of air was administered to the lungs to partly re-inflate them and the rib cage was laterally incised with scissors taking care not to damage the lung tissue.

After the thymus was removed, the heart was twisted slightly to expose the pulmonary artery and then stretched down using a Halstead’s artery clamp (Scientific Laboratory Supplies). An incision was made and the pulmonary artery was cannulated using a cannula similar to the tracheal one. This was secured with a micro aneurysm clip (Harvard apparatus Ltd). Lungs were perfused using a single pass constant flow rate of 8 mL/min. The perfusate was a modified Krebs-Ringer solution (NaCl 118 mM, KCl 4.7 mM, CaCl_2_ 2.5 mM, MgSO_4_ 1.2 mM, NaHCO_3_ 24.9 mM, KH_2_PO_4_ 1.2 mM, HEPES 10 mM, D-glucose 11 mM, 4.5% *w*/*v* BSA, heparin 35 kU/mL, pH = 7.4) maintained at 37°C and saturated with 95% O_2_ and 5% CO_2_. The pericardium was dissected free to allow free efflux of the perfusate and lungs were inflated manually with 1.5 mL air using a 10 mL syringe connected to the intratracheal cannula. As soon as the tissue blanched, the lungs were removed carefully from the chest cavity while maintaining the perfusion and a semi micro Rexaloy clamp (Fisher Scientific, Loughborough, UK) was used to suspend the lungs vertically above a funnel and beaker.

### Absorptive Drug Transfer in the Isolated Perfused Lung

The drugs investigated, 200 nM [^3^H]-formoterol, 130 nM [^3^H]-terbutaline, 130 nM [^3^H]-metoprolol, 275 nM [^3^H]-propranolol, 100 nM [^3^H]-imipramine and 65 μM [^14^C]-dextran 10 K solutions, were made up in Hank’s balanced salt solution (HBSS) at concentrations determined according to their specific activity. A paracellular marker; [^3^H]-mannitol 12.5 nM or [^14^C]-mannitol 65 μM to allow dual counting, was added to the test compound solutions as a control of the lung barrier properties.

After isolation, the lungs were allowed to stabilize for 1–2 min. The syringe attached to the intratracheal cannula was then disconnected and 100 μL of the test solutions were instilled into the airways using a Hamilton microsyringe. Lungs were re-inflated with 1.5 mL of air and sampling was performed by collecting the effluent solution dripping from the left atrium at different time intervals for 90 min. Lung viability was assessed by visual inspection for any sign of oedema as well as by the profile of mannitol airway to perfusate transfer. Samples were assayed by liquid scintillation after addition of 5 mL of Ready Protein ^+®^ scintillant using a 1209 Rackbeta dual scintillation counter.

The cumulative percentage of drug transferred from the airways to the perfusate in 90 min was calculated as the fraction of the administered dose recovered in the perfusate. The time needed for 50% of the drug recovered after 90 min to pass into the perfusate was defined as the absorption half-life (t_1/2_ abs) [19]. The apparent first-order absorption rate constant (Ka_IPL_) was calculated as follows:

$$ {\mathrm{Ka}}_{\mathrm{IPL}}=\frac{\ln 2}{t\raisebox{1ex}{$1$}\!\left/ \!\raisebox{-1ex}{$2$}\right. abs} $$

Absorption data were collected using 4 or 5 IPL preparations.

### Cell Culture

Calu-3 cells were purchased from the American Type Culture Collection (ATCC, Rockville, USA) and 16HBE14o- cells were a gift from Dieter Gruenert (California Pacific Medical Center, San Francisco, USA). Normal human bronchial epithelial (NHBE) cells (Clonetics™, 1st passage) and bronchial epithelial cell growth medium (BEGM) bullet kit were obtained from Cambrex BioScience, Inc. (Walkersville, MD, USA).

Calu-3 cells (passages 26–31) were grown in Dulbecco’s modified Eagle’s medium (DMEM) nutrient mixture F-12 Ham supplemented with 10% foetal bovine serum, 100 UI/mL penicillin, 100 μg/mL streptomycin, 20 mM L-glutamine and 1% *v*/v non-essential amino acids. For solute permeability experiments, cells were seeded onto 24-well polyester Clear Transwell^®^ cell culture inserts (0.4 μm pore size, 0.33 cm^2^ surface area, Costar Corning) at a density of 100,000 cells/cm^2^. After 24 h in culture, the medium was removed from the apical compartment to allow cells to grow at an air-interface as described previously [15]. Cell layers were used after 10–14 d in culture.

16HBE14o- cells (passages 31–33) were cultured in Minimum Essential Medium (MEM) supplemented with 10% foetal bovine serum, 100 UI/mL penicillin, 100 μg/mL streptomycin, 20 mM L-glutamine and 1% *v*/v non-essential amino acids. They were seeded onto 24-well polyester Clear Transwell^®^ cell culture inserts (0.4 μm pore size, 0.33 cm^2^ surface area) at a density of 2.5 x 10^5^ cells/cm^2^ and were grown as described previously [12] for 7 d before drug transport studies.

NHBE were cultured in a cell culture flask using the BEGM bullet kit provided by the supplier until reaching 70–80% confluence. They were then seeded onto 12-well polyester Clear Transwell^®^ cell culture inserts (0.4 μm pore size, 1.13 cm^2^ surface area) at a density of 2.5 x 10^5^ cells/cm^2^ in serum-free BEGM:DMEM/F12 Ham 1:1 supplemented with hydrocortisone (0.5 mg/mL), insulin (5 mg/mL), transferrin (10 mg/mL), epinephrine (0.5 mg/mL), triiodothyronine (6.5 mg/mL), gentamicin (50 mg/mL), amphotericin-B (50 mg/mL), retinoic acid (0.1 ng/mL), and epidermal growth factor (0.5 ng/mL human recombinant) [20,21]. After 24 h, cells were cultured at an air interface. Cell layers were used for transport studies after 14 d in culture.

All cells were maintained in a 5% C0_2_, 95% air atmosphere at 37°C and provided with fresh medium every 2–3 d (Calu-3 and 16HBE14o-) or every 1–2 d (NHBE). Development of confluent cell layers with suitable tight junctions was monitored by transepithelial electrical resistance (TER) measurement using an epithelial VoltOhmMeter (World Precision Instruments, Stevenage, UK) with silver chloride chopstick electrodes.

### *In Vitro* Drug Permeability Measurements

Drugs were presented in HBSS for transport studies in Calu-3 and NHBE or in serum-free medium for transport studies in 16HBE14o-. Non-radiolabelled compounds were added to the test solutions to reach a total drug concentration of 10 μM; solutions were buffered at pH 7.4. ^3^H– or ^14^C–labelled mannitol was added to the solution to produce a 10 μM concentration of paracellular marker to serve as an internal standard for cell layer integrity.

All solutions used in the transport experiment were pre-warmed to 37°C. In preparation for transport experiments, cell layers were washed twice with HBSS (Calu-3, NHBE) or serum-free medium (16HBE14o-). After 30 min equilibration, the pre-experiment TER of each monolayer was measured. The resistance of the cell-free culture support was subtracted from the gross resistance to yield the TER of the epithelial cell layers. Cell layers with TER > 200 Ω cm^2^ were used in transport experiments and TER was monitored to remain within 10% of the initial value over the course of experiments. Drug transport was measured in the absorptive apical (A) to basolateral (B) direction. To initiate the transport measurements, test solutions were added to the apical donor chamber and cell culture supports transferred into a base plate containing HBSS (Calu-3 and NHBE cells) or medium (16HBE14o-) supplemented with 1% bovine serum albumin. Within 1 min, 10 μL of the test solution was removed from the donor chambers to establish the initial donor concentration (C_o_). Cell layers were placed in a 37°C incubator on an orbital shaker rotating at 100 rpm. Every 30 min for 2 h, cell inserts were carefully removed from the basolateral chambers and transferred to a fresh base plate containing pre-warmed transport medium. At each time point, 500 μL were sampled from the receiver compartments. Between samples, the cell layers were returned to the 37°C incubator. After 2 h, 10 μL of sample was removed from the apical chamber to determine the final donor concentration and the post experiment TER was measured.

Samples were analysed by liquid scintillation counting, after addition of 5 mL of Ready Protein^+®^ scintillant using a 1209 Rackbeta dual scintillation counter. Apparent permeability coefficient (P_app_) were calculated using the following equation:

$$ {\mathrm{P}}_{\mathrm{app}}=\left( dQ/ dt\right)/\left({\mathrm{AC}}_{\mathrm{o}}\right) $$

Where *d*Q/*d*t is the transport rate; A is the surface area of the cell culture support, and C_o_ the initial drug concentration in the donor chamber. Transport data were obtained from 6 cell layers from 2 different passages in Calu-3 and 16HBE14o- and from 3 cell layers from passage 2 in NHBE.

### Statistical Analysis

Differences in IPL drug permeability data were compared using Kruskal-Wallis non-parametric ANOVA. Relationships between IPL transport parameters, *in vivo* absorption data in rats and *in vitro* solute permeability in airway cell layers were analyzed using the Spearman’s correlation coefficient. The statistical analysis was performed using SPSS 14.0 for Windows software (SPSS Inc., Chicago, Illinois, USA).

## Results

### Absorptive Drug Transfer in Isolated Perfused Lungs

**Fig. 1:**
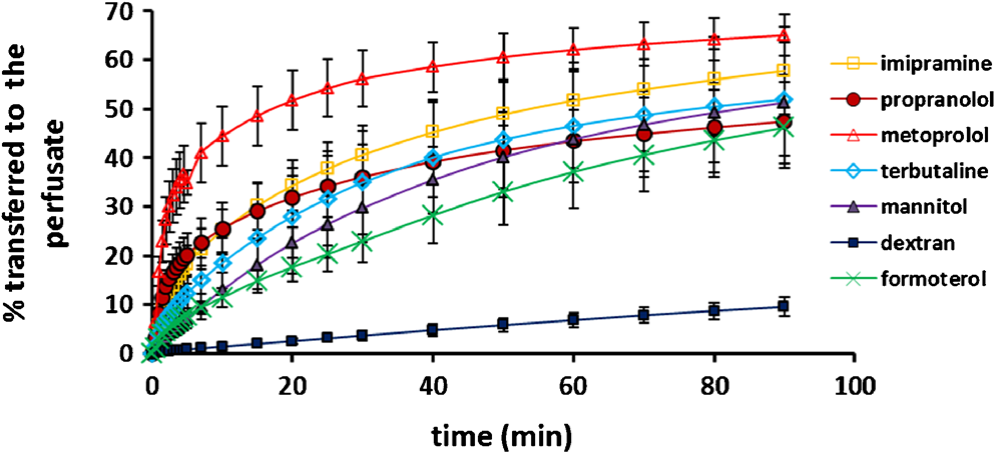
Cumulative percentage of initial dose transferred to the perfusate *vs* time profiles (data not fitted) after intratracheal instillation to isolated perfused rat lungs. Dextran = dextran 10 K. Data are presented as mean ± SD (*n* = 4 or 5).

Following instillation of test solution using a micro-syringe, absorptive solute transfer into the perfusate was measured as cumulative % transferred over 90 min (Fig. 1). Transfer of the high molecular compound, dextran 10 K, was linear with only 9.5 ± 1.9% transported at 90 min (Table II), demonstrating that the epithelial barrier of the lung was maintained over the duration of the experiment in the simple IPL model. The mean proportion of the low molecular weight compounds transferred ranged between 46–65% (Table II). As the cumulative drug transferred to perfusate either reached a plateau (metoprolol and propranolol) or was approaching a plateau (other small molecules) (Fig. 1), this appeared to represent the proportion of dose available for transfer under the experimental conditions. It is interesting that four of the small compounds were not fully absorbed after 90 min; this is most likely related to retarded transport kinetics specific to the properties of the individual compounds, i.e. tissue binding of small basic compounds, although redistribution after instillation to regions of the lungs from which absorption in the IPL can occur may play some role.

**Table I:** Panel of Test Compounds for Permeability Evaluation

Compound	Log P	MW (Da)	Ka _*in vivo*_ (min^−1^)
Dextran 10 K	-	10,000	0.00007 ^(2)^
Mannitol	−3.1	182	0.011 ^(3)^
Terbutaline	0.1	274	0.06 ^(1)^
Formoterol	1.1	840	0.19 ^(1)^
Metoprolol	1.9	684	0.58 ^(1)^
Propranolol	3.0	259	-
Imipramine	4.4	316	0.53 ^(1)^

*In vivo* Ka data are from (1) ref. [22], (2) ref. [23] and (3) ref. [24]

**Table II:** Absorptive Transfer of Compounds after Intratracheal Delivery to the IPL. Data are Presented as Mean ± SD

Compound	n	Ka_IPL_ (min^-1^)	t1/2 abs (min)	% transferred to the perfusate in 90 min
Dextran 10 K	5	0.017 ± 0.002	41 ± 6	10 ± 2
Mannitol	5	0.029 ± 0.005	25 ± 5	51 ± 4
Terbutaline	4	0.037 ± 0.006	19 ± 3	52 ± 12
Formoterol	4	0.023 ± 0.002	30 ± 2	46 ± 7
Metoprolol	4	0.260 ± 0.050	3 ± 1	65 ± 4
Propranolol	4	0.098 ± 0.016	7 ± 1	47 ± 9
Imipramine	4	0.071 ± 0.021	10 ± 3	58 ± 9

**Fig. 2:**
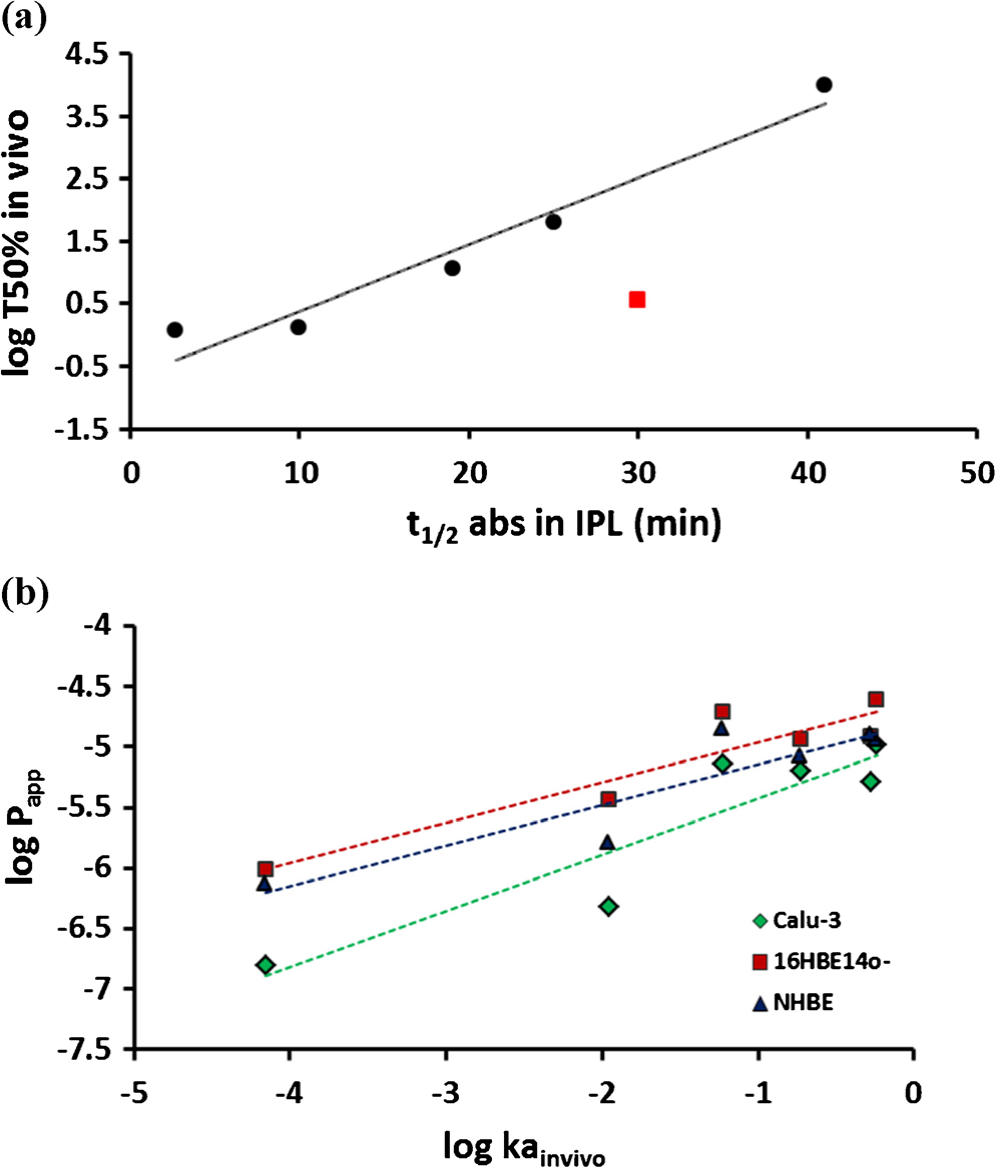
Relationship between pulmonary absorption *in vivo* in rats and absorption / permeability in (**a**) isolated perfused rat lungs - half-time of solute absorbed in 90 min in the IPL (t_½ abs IPL_). Formoterol (square on the plot) has been excluded from the correlation. (**b**) human airway epithelial cell layers - the apparent permeability coefficient (logarithm of Papp; cm/s) in cell culture absorption models based on the Calu-3 and 16HBE14o- cell lines and normal human bronchial epithelial cells (NHBE).

The more lipophilic compounds imipramine, metoprolol and propranolol were transferred into the perfusate faster than the hydrophilic compounds as evident from the absorptive profiles (Fig. 1) and t_1/2_ (Table II). The data were analysed using the approach of Tronde *et al*. [19] for simplicity and to enable comparison with the published data. This approach includes an assumption of first order kinetics, although the full cumulative absorptive transfer profile is not utilized for compounds which do not reach a plateau in 90 min, thus the Ka_IPL_ parameter may be misleading. However, differences in absorptive flux of the test compounds were clearly apparent and the calculated parameters provided a ranking reflective of transfer profiles in the first 5–10 min, where the greatest differences in solute transfer to the perfusate were observed. Absorptive drug transfer in the IPL was compared with reported pulmonary absorption data from rat lungs [22–24]. A strong relationship was observed between the logarithm of the *in vivo* absorption half-life (log T_50%_) and the absorptive half-time (t_½_ abs) in the IPL (*r* = 0.97, *p* < 0.01, Fig. 2a) when formoterol, which appeared as an outlier, was excluded from the analysis.

### Drug Permeability in Airway Epithelial Cell Layers

**Fig. 3:**
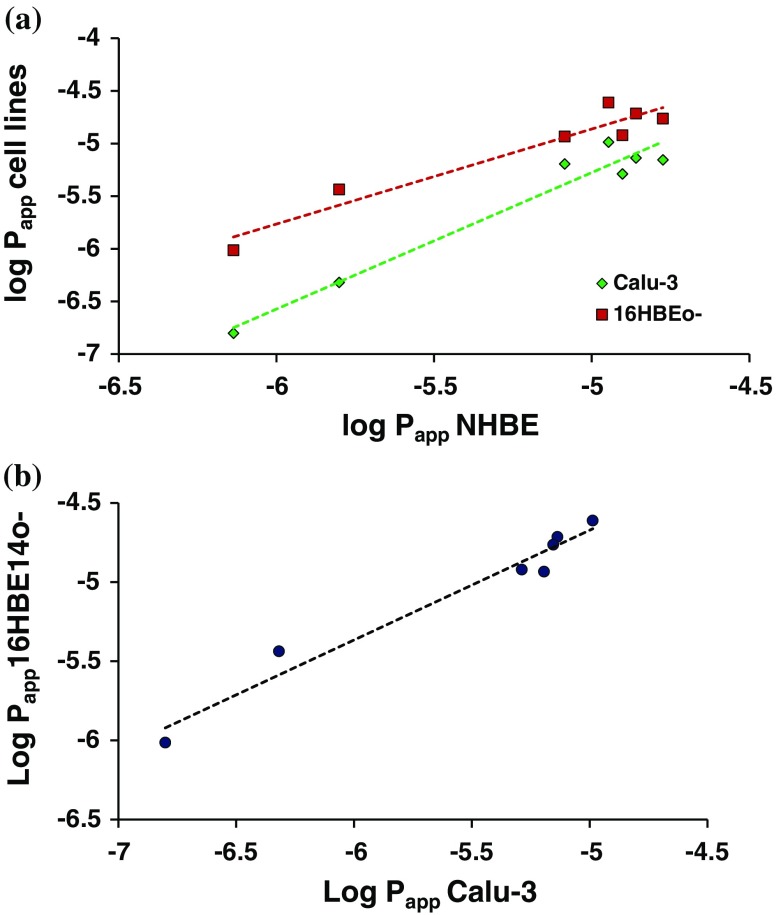
Comparison of permeability measured using *in vitro* methods. (**a**) The relationship between apparent permeability (logarithm P_app_; cm/s) in the Calu-3 and 16HBE14o- cell lines compared to normal human bronchial epithelial cells (NHBE). (**b**) Correlation between the apparent permeability (P_app_) in Calu-3 and 16HBE140- cell layers.

The absorptive permeability of each compound was also measured in the airway epithelial cell drug transport models. The cell lines produced cell layers with TER and P_app_ of mannitol within the normal range for these models [4]: Calu-3: TER = 280 ± 10 Ω cm^2^, P_app_ = 0.48 ± 0.06 x 10^−6^ cm s^−1^; 16HBE14o-: TER = 240 ± 20, P_app_ = 3.7 ± 0.5 x 10^−6^ cm s^−1^. The primary cell layers produced similar resistances and permeability to mannitol: NHBE: TER = 330 ± 110 Ω cm^2^, P_app_ = 1.6 ± 0.5 x 10^−6^, cm s^−1^. The recovery (mass balance) of compounds was >70% (except for imipramine in NHBE for which recovery was 56.5 ± 2.9%; losses likely due to binding to plasticware and drug in the cellular compartment) and the cumulative drug transported *vs* time profiles were linear in all instances (R^2^ > 0.98). In each of the cell culture models, the permeability of the hydrophilic molecules mannitol and dextran 10 K, which permeate cell layers exclusively via the tight junctions and serve as paracellular markers, was lower than that of the more lipophilic therapeutic molecules. The permeability of the cell layers to the paracellular markers ranked Calu-3 > NHBE >16HBE14o- (Table III). The rank order of permeability for the compounds investigated was identical in Calu-3 and 16HBE14o- layers, but varied for the more lipophilic compounds in NHBE cell layers (Table III). Log linear relationships between drug permeability in the cell layers *in vitro* and the apparent first order absorption rate constant *in vivo* were observed (*r* = 0.92, 0.93 and 0.91 in Calu-3, 16HBE14o- and NHBE cells, respectively; Fig. 2b, p < 0.05). Even stronger log-linear relationships were obtained between solute permeability measured in the different *in vitro* models, i.e. *r* = 0.97 for Calu-3 and 0.96 for 16HBE14o- *vs* NHBE (Fig. 3a) and *r* = 0.98 for Calu-3 *vs* 16HBE14o- (Fig. 3b).

**Table III:** *In vitro* Permeability in Airway Cell Layers. Data Represent Mean ± SEM (*n* = 6, *n* = 36 for Mannitol) in Calu-3 and 16HBE14o- and Mean ± SD (*n* = 3, *n* = 18 for Mannitol) in NHBE

	P_app_ (10^−6^ cm/s)		
	*Calu-3*	*16HBE14o-*	NHBE
Dextran 10 K	0.2 ± 0.0	1.0 ± 0.1	0.7 ± 0.1
Mannitol	0.5 ± 0.1	3.7 ± 0.5	1.6 ± 0.5
Terbutaline	7.3 ± 0.4	19.3 ± 2.0	13.8 ± 0.8
Formoterol	6.4 ± 0.3	11.7 ± 0.8	8.2 ± 0.8
Metoprolol	10.3 ± 0.0	24.5 ± 2.5	11.3 ± 0.7
Propranolol	7.0 ± 0.4	17.3 ± 0.8	16.8 ± 3.0
Imipramine	5.2 ± 0.0	12.0 ± 0.9	12.5 ± 0.9

### *In Vitro –Ex Vivo* Correlations

**Fig. 4:**
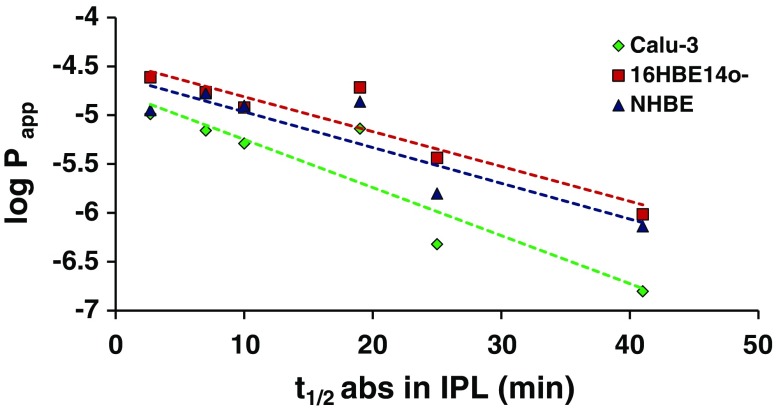
Comparison of permeability measured using *in vitro* and *ex vivo* methods. The relationship between absorption half-life in the IPL (t_½ abs IPL_) and permeability in airway epithelial cell layers (logarithm P_app_; cm/s). Formoterol has been excluded from the correlation.

Linear relationships were obtained between the logarithm of the P_app_ in cell layers and the absorption half-life in the IPL when formoterol, an outlier in the IPL-*in vivo* correlation, was excluded from the analysis. These relationships were stronger for the cell lines compared to the primary cell model (*r* = 0.92 for Calu-3 cells; *r* = 0.93 for 16HBE14o- cells; *r* = 0.89 for NHBE cells; Fig. 4, *p* < 0.01). This was similar to previous evaluations comparing solute permeability in 16HBE14o- cell layers [17] and Calu-3 cell layers [16] with drug transfer/absorption from the lungs.

## Discussion

A recent AAPS/FDA/USP workshop considered a systematic framework to classify pulmonary drugs to provide a tool for formulators and discovery chemists working in the pulmonary drug delivery field [6]. Drug permeability or the rate of absorption was identified as an important predictor of local residence time and, therefore, duration of effect for locally-acting drugs. When drugs are administered to the lungs for the purpose of systemic delivery, drug permeability is a critical determinant of bioavailability. If pulmonary drug permeability and the rate of absorption are to be utilized in an iBCS, there is requirement for simple and reliable methods to screen inhaled drug candidates for this property at an early stage of their development.

The IPL preparation has been explored as an *ex vivo* model for screening the pulmonary absorption of drugs by AstraZeneca [19] and GlaxoSmithKline [25]. There are a number of methodological variations in how the IPL technique is configured [5], and a factor limiting the wider adoption of the IPL as a drug absorption model has been the perceived requirement for sophisticated systems for delivering drugs and maintaining the organ preparation. In contrast, the approach taken in this study was to evaluate the minimum requirements, i.e. the simplest system, that will permit the lungs to be used *ex vivo* to obtain absorptive drug transport data. A low-cost, simple IPL model requiring no specialist equipment in which the lung viability was maintained for more than 90 min was developed. This is within the 20–120 min duration over which airspace-to-perfusate drug transfer has been reported [19,25]. Drug administration by instillation was adopted for simplicity; and is favoured by some investigators [25] although fine sprays [19] and aerosol administration [26] have been used, but introduce complexity. In this study, the profiles of drug transfer from the airspaces to the perfusate (Fig. 1) were comparable to those reported after administration of drug by nebulizer catheter to a more complex physiologically-controlled IPL system [19], and a similar correlation with pulmonary drug absorption *in vivo* was obtained. A number of different systems are available for delivering aerosols to the IPL, e.g. the PreciseInhale^®^ system from Inhalation Sciences, which is an important aspect if aerosol formulation-driven absorption kinetics are to be studied. Although instillation may not penetrate the airways as fully as aerosol administration, this method of delivery gives precise control over dosimetry and allows discrimination between the absorptive transport of drugs on the basis of their physicochemical characteristics [25].

The β_2_-adrenoreceptor agonist formoterol appeared as a poorly transported outlier in the IPL/*in vivo* correlation; if formoterol is excluded from the analysis, the correlation is *r* = 0.97. By contrast, in a study by Tronde *et al*., formoterol fitted well with the IPL/*in vivo* linear relationship obtained [19], although both studies suffered from low numbers of compounds for *in vitro-in vivo* correlation. The airway absorption of inhaled β_2_-adrenergic agonists is complex. Although formoterol has relatively low lipid solubility due to a net positive charge, lung tissue retention is observed due to high levels of tissue binding [27]. Formoterol charge is highly pH dependent over the range pH 6–8 (speciation *vs* pH plot [28]) which can influence both passive permeability and interaction with the pH-dependent cation transporters that transport formoterol in the airway [29]. It is possible that small pH changes in the lung lining fluid may have occurred, pH was not measured and it would be interesting to study in detail the effect of pH changes with regard to formoterol transport in the IPL.

IPL has become sufficiently well established and valued for drug permeability screening that it has been used to generate quantitative structure activity relationship (QSAR) [25]. Quality control and validation of the IPL system is important if the model is to be useful as a screen for pulmonary drug absorption, and used to evaluate transport mechanisms, including the effects of drug transporters [30]. Thus, it will be important to establish benchmarks, controls and acceptance criteria for the technique, which may need to be specific for different applications. The data presented herein provides a proof-of-principle that the simple IPL provides useful drug transport data, but is limited by the modest number of compounds evaluated. To establish definitively that the technique is predictive of pulmonary drug absorption would require a larger range of compounds (for which *in vivo* data is available), separated into a probe set to establish a predictive model and a test set with which to test it.

The benefit of the simplified IPL described in this study is that it avoids the need for specialized equipment and requires only the skills of an *in vivo* pharmacologist to isolate and maintain the lungs *ex vivo*. The technique is not in itself sparing of the use of animals in research and it is more costly and has lower capacity than cell culture. However, it is a method under which lung processes, such as absorption or metabolism, can be isolated and studied in a system which preserves three-dimensional organ architecture under carefully controlled conditions, enabling studies to obtain answers with fewer replicates by avoiding interference from systemic influences. In addition to measuring the intrinsic permeability of drugs, the IPL technique is being used to evaluate the effectiveness of a variety of absorption-modifying drug delivery strategies on absorptive clearance from the lungs, including nanoparticles [31], sequence-specific phage display-derived peptide conjugated dendrimers [32], drug-ester polymer conjugates [33], liposomes [34] and polymer microparticles [35].

Drug permeability in respiratory epithelial cell lines is well-correlated with the pulmonary absorption rate constant in rats [12,16], which makes the cell lines useful for rank-ordering and screening drugs with respect to their intrinsic lung absorption rates [6]. We compared directly the permeability of seven molecules in Calu-3, 16HBE14o- and NHBE cell layers, deriving log-linear relationships between their permeability in Calu-3, 16HBE14o- or NHBE cell layers and the absorption rate constant determined after pulmonary delivery to rats (Fig. 2b). Higher apparent permeability coefficients were obtained in 16HBE14o- and NHBE compared to Calu-3 cells, whereas the same rank order was obtained in the cell lines, 16HBE14o- and Calu-3. In terms of molecular properties, the molecules with log *P* > 0.1 possessed higher permeability and were clustered with Papp values in the range 5.2–24.5 x 10^−6^ cm.s^−1^ in all the cell models. The Calu-3 cell layers were more restrictive to the large molecule, dextran MW 10,000, and hydrophilic small molecule, mannitol. Strong *in vitro/* *in vivo* correlations have been reported previously with compounds possessing a wider range of molecular weight, and therefore a wider range of permeability, i.e. dextrans MW 4000, 10,000, 40,000 and 70,000 [16]; in contrast dextran 10,000 was the only non-small molecule in the data sets reported herein.

When selecting *in vitro* models for pre-clinical screening, there is generally a trade-off between practicalities (simplicity, economy, reproducibility, capacity) and biorelevance (human systems, mixed cell types, structural/morphological/dynamic features). Although NHBE cells provide a more biorelevant model, for routine use this advantage is outweighed by the convenience, low cost and robustness of cell lines, especially if no advantage of the primary cell model can be rationalized or demonstrated. For the compounds used in this study, *in vitro* permeability correlated with *in vivo* absorption for each of the cell models, with no advantage apparent in regard of utilizing one cell model over another. Although comparison of compound permeability in the respiratory cell-based models showed strong relationships with absorption from the IPL (Fig. 4) and rat lungs *in vivo* (Fig. 2b), a similar relationship has been reported between permeability in Caco-2 cells and absorptive transfer in IPL [19]. For other aspects of lung absorption/retention, e.g. drug transport mechanisms, lung-targeting strategies and the efficacy of inhaled medicines, more organ-specific models may be required with requirements which should be carefully considered on a case-by-case basis for each application [7]. If respiratory cell lines are to be used to generate decision-making data, e.g. for selecting compounds for development as orally inhaled products, similar principles to those advocated for the use of Caco-2 in predicting intestinal drug permeability should be applied to maximise data quality, i.e. standardized practices for culture of cells, conduct of experiments, use of benchmarks and data analysis [36].

## Conclusion

The physicochemical properties of molecules that confer good biopharmaceutical performance when inhaled are not fully understood [6,37,38]. *In vitro* and *ex vivo* techniques provide experimental models in which drug permeability in the lungs can be derived empirically. Of the techniques available for pre-clinical characterization of drug permeability, a hierarchy ranging from in silico methods to *in vivo* studies transport has been proposed previously [5]. Under such a model, cell cultures should be used for initial screening of drug permeability before proceeding to *ex vivo* and *in vivo* techniques for lead candidate optimization. Interestingly, non-cellular PAMPA methods have been developed for certain epithelia, e.g. intestinal and blood brain barrier [39], but not to date for lung permeability measurements. In this absence, cell lines provide an opportunity to reduce animal testing and can be used to determine intrinsic drug permeability in drug design and development [40] and generate essential inputs for mechanistic modelling. In our study, drug permeability in a much simpler IPL method than previously reported was indicative of *in vivo* lung absorption and concorded with findings in the cell culture models. All of the techniques were suitable for empirical screening of drug absorption in the lungs, with Calu-3 out of the cell models having the advantage for routine drug permeability screening purposes of being commercially available and more robust in forming tighter air-interfaced cell layers compared to 16HBE14o- cells and more economic and simpler to culture than NHBE.

## Abbreviations

AAPS: American association of pharmaceutical scientists
P_app_: Apparent permeability coefficient
BEGM: Bronchial epithelial cell growth medium
DMEM: Dulbecco’s modified eagle’s medium
FDA: Food and drug administration
HBSS: Hank’s balanced salt solution
IPL: Isolated perfused lungs
iBCS: Inhalation biopharmaceutical classification system
MEM: Minimum essential medium
MW: Molecular weight
NHBE: Normal human bronchial epithelial
QSAR: Quantitative structure activity relationship
TER: Transepithelial electrical resistance
USP: United States pharmacopoeia
